# Reuse of treated water in European agriculture: Potential to address water scarcity under climate change^☆^

**DOI:** 10.1016/j.agwat.2021.106872

**Published:** 2021-05-31

**Authors:** Jordan Hristov, Jesus Barreiro-Hurle, Guna Salputra, Maria Blanco, Peter Witzke

**Affiliations:** a European Commission, Joint Research Centre (JRC), Seville, Spain; b CEIGRAM, Universidad Politécnica de Madrid, Spain; c European Centre for Agricultural, Regional and Environmental Policy Research (EuroCARE), Bonn, Germany

**Keywords:** Treated water, CAPRI model, Climate change, Water scarcity, Water stress

## Abstract

The use of reclaimed or treated water from urban wastewater treatment plants for irrigation has been proposed as an alternative water source to address water scarcity issues in Europe. In this paper using agro-economic modelling, we analyse if treated water available for agriculture has the potential to reduce freshwater abstraction and, consequently, water stress. Implementing exogenous treated water quantities as an additional water supply at NUTS 2 level in the CAPRI model, we found that treated water reuse is a possible alternative supply source to address water shortages with a very negligible effect on farmers’ income and food production in the EU. However, the actual water reuse and water stress reduction is very limited due to high costs. Even climate change effects on water availability and precipitation failed to induce higher use. The one-size-fits-all approach modelled via a flat rate water price only encourages the reuse of treated water in a limited number of EU member states. Thus, in order to maximise the potential of reused water to address water scarcity, different rates should be used so as to ensure higher treated water volumes at lower costs.

## Highlights

• Water reuse is a possible alternative supply source to address water shortages.
• Negligible effect on farmer's income and food production in EU.
• Actual water reuse and water stress reduction is very limited.
• Financial incentives in form of subsidies necessary to improve water reuse for irrigation.

## 1 Introduction

Water scarcity is already a recurrent problem in some European countries (EEA, 2018a), leading to environmental and economic consequences which will be aggravated in the future due to climate change (Collins et al., 2009). Due to increased temperatures driven by climate change, water availability will be impacted due to increased evapotranspiration and thus changes in precipitation and river flows. Such changes will also affect the capacity of different economic sectors to access water. Simultaneously, water demand is expected to increase due to population growth that will trigger higher use across the sectors. In particular, it is expected that agricultural water use will be intensified to satisfy increased food demand (European Commission, 2018a). It is estimated that in the future more farmland area will have to be irrigated, especially in southern Europe, putting additional pressure on the existing water stress in Europe (EEA, 2018b). Furthermore, there will be an increase in demand by households, tourism and industry leading to a more intense competition for water use. To guarantee that the increased demand and reduced supply do not put at risk the competitiveness and the efficient functioning of the European internal market, all sectors need to improve their water use efficiency. To deliver its share of the joint effort, sustainable irrigation water management is required to mitigate the water deficit in agriculture.

To promote sustainable water management the European Commission (EC) urges farmers, under the current Common Agricultural Policy (CAP), to meet specific agri-environmental targets for water through the cross-compliance mechanism (European Commission, 2019a). In addition, CAP rural development measures support investments for improving the state of irrigation infrastructures or irrigation techniques leading to efficiency gains and a reduction in water losses (*ibid.*). However, the efficiency of such measures may be limited (Berbel et al., 2018, Soto-García et al., 2013). Despite these efforts, the key challenges in terms of water scarcity remain and further actions are needed (EEA, 2018a). Uneven implementation of water abstraction measures across Europe were also identified as a gap in the implementation of the Water Framework Directive (WFD) (European Commission, 2019b). Therefore, other policy measures that can reduce the net use of water in the agricultural sector have been put in place. Recently, the European Council adopted for a regulation which will facilitate the safe reuse of treated urban wastewater (treated water) for agricultural irrigation from urban wastewater treatment plants (European Council, 2020a). The regulation provides a harmonised approach regarding minimum requirements on reference pathogens and risk management when irrigating with reused water in the EU as a climate change adaption tool, which will contribute to lowering water extraction from agriculture together with irrigation modernisation plans that can be included in the CAP beyond 2020. It should be noted that water reuse as an alternative supply option to address EU water scarcity was already acknowledged in the 2012 Commission communications ʻA Blueprint to Safeguard Europe's Water Resourcesʼ (COM(2012) 673) and ʻClosing the loop – An EU action plan for the circular economyʼ (COM(2015) 614).

Bixio et al. (2006) found that in Europe there has been an increase in treated water use for irrigation, especially in southern Europe (Alcon et al., 2012). Lazarova et al. (2001) reviewed the role of treated water and its contribution to integrated water management. It was concluded that treated water is a reliable alternative resource which prevents the degradation of the environment. Barbagallo et al. (2012) have also found that treated water is a potential alternative irrigation source and has a fundamental opportunity to reduce freshwater use and improve the environment as well as the agricultural sector. Pedrero et al. (2010) have similar findings. However, in their studies an economic assessment is lacking and is also limited to specific case studies (Italy, Spain, Greece, France). In an economic evaluation by Vergine et al. (2017), it was found that treated water reduces the stress on conventional resources, especially during the drier seasons. Furthermore, Alcon et al. (2012) show that the implementation of treated water in the context of the WFD produces significant environmental benefits that are greater than the investment and operational costs of the treatment plants. Nevertheless, despite the economic assessment, the findings are limited to Italy and south-eastern Spain. Pistocchi et al. (2017) found that farmers’ attractiveness of using treated water for irrigation vary depending on the infrastructure costs and the distance from the treatment plant to the irrigated land. Therefore, there is still a knowledge gap concerning how additional availability from reused water at a given cost will be adopted by the agricultural sector at EU level and how these costs impact agricultural and food production and prices of agricultural commodities in the EU. This is particularly relevant if the full price recovery principle of the WFD is implemented. It is also unclear how freshwater abstraction will change and if water stress will be reduced at EU level. Thus, the aim of this paper is to provide insights into whether water reuse is a viable alternative to reduce the risk of water shortages for irrigation in the EU and how it can contribute to mitigating water scarcity across the EU in the context of adapting to climate change. If this is the case then it would also contribute to the reduction in freshwater abstraction, leading to a reduction in water stress.

To answer these questions, we use the Common Agricultural Policy Regional Impact Analysis (CAPRI) model with its updated water module (Blanco et al., 2018). The CAPRI water module was developed with the purpose of making simulations of the potential impact of water availability on agricultural production in the EU (Blanco et al., 2015). It integrates detailed water considerations of CAPRI including crop-specific irrigation water use and livestock water use as a specific input in production at Nomenclature of Territorial Units for Statistics (NUTS) 2 level. Thus, being able to capture such food-water linkages makes the CAPRI water modelling framework suitable for this purpose. The study contributes to the existing literature on the reuse of treated water in agriculture in two main ways: first, it considers the potential impact on production using novel estimates of the availability of treated water at NUTS2 level. Second, it evaluates the role of treated water considering the reduction on water availability due to climate change.

The rest of the paper is structured as follows. In Section 2 we present the modelling approach, followed by the scenario definition in Section 3. Section 4 covers the simulation results, and in the final Section 5 we discuss the results of this study and highlight the main conclusions.

## 2 Methodology

To achieve the aim of this research we use the agro-economic CAPRI model with its water module. CAPRI is a partial equilibrium, large-scale economic, global, multi-commodity, agricultural sector model (Britz and Witzke, 2014). The effects of agricultural, environmental and trade policies on agricultural production, farm prices and income, trade as well as environmental indicators are analysed in a comparative-static framework where the simulated results are compared to a baseline scenario that is calibrated to the 2017 Agricultural Outlook published by the EC (European Commission, 2017), which considers the continuation of the CAP 2014 up to 2030.

CAPRI consists of a supply module for the EU (at regional, member state (MS) and aggregated EU level) that interacts with a global market module where bilateral trade and prices for agricultural commodities are computed. The supply module covers more than 50 inputs and outputs which are produced or used in more than 50 crop and livestock activities in about 280 NUTS 2 regions within the EU. The production of 47 primary and processed agricultural products from the supply module is covered by 77 countries in 40 trade blocks in the market model, and the two modules interact until equilibrium is reached (Britz and Witzke, 2014). The main simulated results provided by CAPRI are related to crop areas and yields, production, prices, income.

The water module in CAPRI is implemented in the supply part of CAPRI (i.e. for the EU, Western Balkans and Turkey). For the analysis we use the recently updated CAPRI water module (Blanco et al., 2018). Water is included as a production factor in both crop and livestock production. In addition, areas are split into irrigated and rain-fed, considering water use, irrigation efficiency and differentiated crop yields of irrigated and rain-fed activities. It should be noted that some activities in CAPRI are aggregates and the technical coefficients are not purely crop-specific. Instead, for these crop aggregates, the input–output coefficients for the rain-fed/irrigated variants are defined to match the average activity coefficients of that aggregate (Blanco et al., 2015). Irrigated areas are based on the 2010 Survey on Agricultural Production Methods (SAPM) and the 2000–2013 EUROSTAT Farm Structure Survey (FSS) data. Crop-specific and regional irrigation requirements were obtained from the CROPWAT^1^ model and the potential and water-limited yields at NUTS 2 level were derived from biophysical simulations using the World Food Studies (WOFOST) model.^2^ The data from CROPWAT covers a set of 12 crops (soft wheat, maize, paddy rice, sunflower, olives, potatoes, sugar beet, tomatoes, apples, citrus fruits, table grapes and wine production). For the CAPRI crops not directly matched to CROPWAT crop simulations, assumptions were used by assuming that the non-modelled crop has the same value as a ʻsimilarʼ crop (see Appendix A). A similar approach was also applied to the 10-crop set from WOFOST (wheat, barley, rye, maize, field beans, sugar beet, rapeseed, potato, sunflower and rice). The updated module uses data on water use from the Distributed Water Balance and Flood Simulation Model (LISFLOOD). It is based on the 2006 simulations since it is the most complete, consistent, quality and comparable water data set available at the time of the recent update of the water module used for this paper. As mentioned, the updated water module includes water as a production factor in rain-fed agriculture. In the previous version, water-food linkages were limited to irrigated agriculture only. Thus, besides linking treated water to irrigated water availability, we are now able to also link impacts of treated water reuse to those originating from changes in precipitation due to climate change. Farmers are flexible and able to switch production between irrigated and rain-fed alternatives depending on the climate change shock as well as the available treated water. Hence, we are able to provide a better understanding of the treated water reuse potential with respect to climate change. It should be noted that CAPRI does not currently explicitly model deficit irrigation but it is indirectly captured through the yield ratio between potential and water-limited yields derived from the WOFOST model (Blanco et al., 2015). This ratio also equals the rain-fed to irrigated yield ratio which allows the differentiation of rain-fed and irrigated yields and substitution between the crop variants. Therefore, if there are changes in effective rainfall and its distribution over the crop-growing season, the water-limited yield is lower than the potential yield under irrigated conditions, i.e. the irrigated crop variant gains more weight if there is no water availability stress and there will be a substitution between the rain-fed and irrigated crop variant. The opposite also applies if there are irrigation water shortages.

**Fig. 1:**
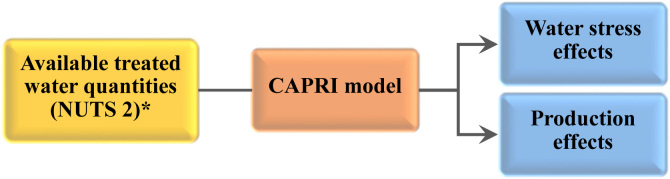
Flowchart of modelling. Note: * Data from Pistocchi et al. (2017). Source: Own illustration.

Data on the potential supply of reused water at different cost levels in EU at NUTS 2 level were taken from Pistocchi et al. (2017) as exogenous additional water availability in the model (Fig. 1). We focus on treated water availability at €0.5/m^3^ total costs. These costs include the treatment cost (€0.08/m^3^) and additional charges such as pumping, transportation, storage and charges for potential expansion and maintenance of the irrigation infrastructure (*ibid.*). Since these costs cannot be separated in the CAPRI water module, we assume that the total cost equates to the water price for extra m^3^ of treated water (full cost recovery). We assume full cost recovery because water supply benefits alone cannot cover the treatment costs (Raso, 2013). The additional cost was modelled to account for simulating water price scenarios (Blanco et al., 2015) where the total cost related to the consumed water quantities, are introduced in the objective function. According to Pistocchi et al. (2017), the available treated water quantities at €0.5/m^3^ treatment costs provide the highest benefits in terms of nutrient load and water stress mitigation. Below the €0.5/m^3^ cost, limited reused water quantities can be provided, and above this threshold the net benefits in terms of cost are significantly lower. To comply with the assumptions in the study by Pistocchi et al. (2017), we consider only quantities that can actually reach the irrigation demand within a 10 km distance from the urban wastewater treatment plants. This means that, in some regions the available quantities were higher than the irrigation demand that can actually be reached. In those few cases/regions in Belgium, Germany, Italy, the Netherlands and Portugal, we considered the irrigation demand rather than the availability from the treatment plants. It should be noted that the additional treated water quantities are available for the entire NUTS 2 regions since it is the smallest spatial unit in CAPRI and cannot apply the 10 km distance criterion. The simulation year is 2030, given the high degree of uncertainty regarding the macroeconomic and agricultural projections over longer periods. We also assume that there will be no change in terms of the distribution of wastewater treatment plants. In order to capture changes in irrigation requirements due to climate change, we applied changes in irrigated area according to FAO (2017) projections. However, to make sure that we fully capture the treated water reuse potential (not limiting the expansion in an irrigated area), we increased the potentially irrigated^3^ area by 30%.

## 3 Simulated scenarios

To capture the potential of treated water reuse we consider three scenarios and three baselines. The scenarios are needed as the current configuration of the water module in CAPRI does not allow for separating water as an input or the input cost by water source nor separating the crop activities by irrigation water source, as it has been done between irrigated and rain-fed crop activities. To solve this modelling limitation, water reuse scenarios, where only the additional quantities of treated water were added as a shock, are compared to different baselines with and without climate change impacts on freshwater and rain availability. Only in this way we can separate the effects of additional treated water supply from those due to climate change. The main characteristics of the scenarios and baselines are described below and summarised in Table 1.

1. Baseline (BASE): business as usual scenario calibrated to mid-term projections for agricultural markets based on the 2017 Agricultural Outlook for 2030 (European Commission, 2017) and considering the CAP 2014–2020 (Gocht et al., 2017). Irrigation costs are included together with the other cost groups and cannot be explicitly distinguished from them, since EU-wide statistics are lacking in terms of irrigation costs. Since the freshwater price in the baseline is not explicitly observed we have to price it at €0.5/m^3^, i.e., equal to the total cost of treated water when running the baseline as well as the other baseline scenarios. If we shock the system as it works when we introduce the treated water quantities with a specific price, we are not just pricing treated water but also freshwater because, as explained above, in the current water module, there is no possibility of separating the irrigation water supply by source or prices. Thus, there is inconsistency between the baseline and alternative scenario. With the assumption of pricing freshwater already in the baseline, we are able to compare and analyse water reuse effects on water stress reduction and agricultural production. Note that some current MS already use treated water for irrigation, and these quantities are not captured in the baseline scenario.

1. a.Treated water reuse (REUSE): this scenario takes the assumptions of BASE and adds additional water availability equal to the treated water quantities for reuse which can be made available at a total cost of €0.5/m^3^ (see Appendix B) priced at full cost recovery. This scenario allows us to examine if water reuse is a cost-effective measure that could reduce freshwater use and thus water stress. A conventional measure of water stress is the withdrawal-to-availability ratio. However, in this case the difference in water use between the baseline and the scenario can be seen as a change in the water stress. As freshwater in the baseline is priced at the same level as the treated water cost, any additional quantity used when the treated water is allocated should be seen as a shift from fresh to treated water use and a reduction in the water stress.

2. Less freshwater availability for irrigation (WAVA): this scenario considers the same settings as BASE, i.e. the CAP 2014–2020, as well as a price of €0.5/m^3^ for the freshwater to be able to do a meaningful analysis, but additionally considers a reduction in water availability for irrigation as a consequence of climate change. We focus on the Representative Concentration Pathway (RCP) 8.5 that corresponds to a high greenhouse gas emissions pathway with very little mitigation (Riahi et al., 2011), thus having a negative effect on the water resources (Fischer et al., 2007). Under these settings we would like to investigate whether the full potential of treated water can really reduce the risk of water shortages. Region-specific data on water availability for irrigation reductions per NUTS 2 region was obtained from Bisselink et al. (2018).

2. a. Less water availability for irrigation and treated water reuse (WAVA REUSE): this scenario takes the assumptions of WAVA and adds additional water availability equal to the treated water quantities for reuse which can be made available at a cost of €0.5/m^3^. To be able to analyse the effect of water reuse independent of the climate change impacts via reduced freshwater availability, the comparisons are made with respect to the WAVA scenario. This comparison implies that the additional water quantity used can be seen as a shift between water sources and a reduction in the water stress.

3. Less rain (RAIN): this scenario again builds on the assumptions under the BASE scenario. But compared to the BASE scenario, changes in effective rainfall are added to the simulation year attributed to climate change. This will only affect rain-fed agriculture but not irrigated agriculture. However, with less rain the available demand for irrigation is expected to increase with an associated increased water use. Similar to scenario 2, we focus on RCP8.5 and implemented changes in effective rainfall obtained from Bisselink et al. (2018).

3. a. Less rain and treated water reuse (RAIN REUSE): this scenario takes the assumptions of RAIN and adds additional water availability equal to the treated water quantities for reuse which can be made available at a cost of €0.5/m^3^. To be able to analyse the effect of water reuse independent of the climate change impacts via reduced rainfall, the comparisons are made with respect to the RAIN scenario. This comparison implies that the additional water quantity used can be seen as a shift between water sources and a reduction in the water stress.

**Table 1:** Scenarios summary.

No	Name	Description	Scenario drivers
1	BASE	CAP 2014 with a water price of €0.5/m^3^.	Water price.
1a	REUSE	Same structure as BASE but added treated water quantities.	Treated water availability and water price.
2	WAVA	CAP 2014 with a water price of €0.5/m^3^ and reduction in freshwater availability for irrigation under RCP8.5.	Freshwater availability and water price.
2a	WAVA REUSE	Same structure as WAVA but added treated water quantities.	Freshwater and treated water availability, water price.
3	RAIN	CAP 2014 with a water price of €0.5/m^3^ and reduction in effective rainfall under RCP8.5.	Effective rainfall changes and water price.
3a	RAIN REUSE	Same structure as RAIN but added treated water quantities.	Effective rainfall changes, water price and treated water availability.

## 4 Results

We discuss the impacts of making treated water available for irrigation, focusing on two main aspects: firstly, from an environmental point of view we see whether treated water reuse is effective in reducing water stress and, secondly, we see how agricultural markets are affected. This analysis is performed for the three comparisons mentioned above.

### 4.1 Effect on water stress

**Fig. 2:**
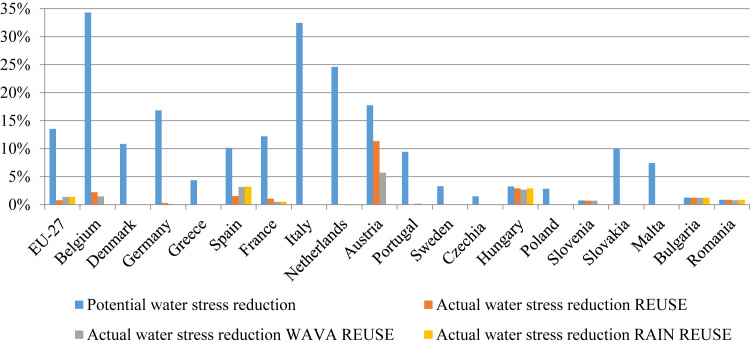
Potential and actual reduction (relative changes to respective baseline) in water stress at regional level in 2030 due to water reuse in REUSE (2a), WAVA REUSE (2b) and RAIN REUSE scenario (2c). Note: Some Member States are omitted from the figure and later in the regional results because no treated water quantities are available. Source: Own elaboration.

As argued in the introduction, the treated water reuse potential in terms of reducing freshwater net use in the agricultural sector, and consequently reducing regional water stress, is noteworthy. Recall that due to the impossibility of separating water sources, the water stress indicator is calculated as the difference between the base and alternative scenario given that the price for water is equal in both scenarios, rather than the conventional method of withdrawal-to-availability ratio. Fig. 2 shows that water stress can be reduced by around 14% on average in the EU, ranging between 35% in Belgium and 1% in Slovenia, the Czech Republic, Bulgaria and Romania (Fig. 2).

**Fig. 3:**
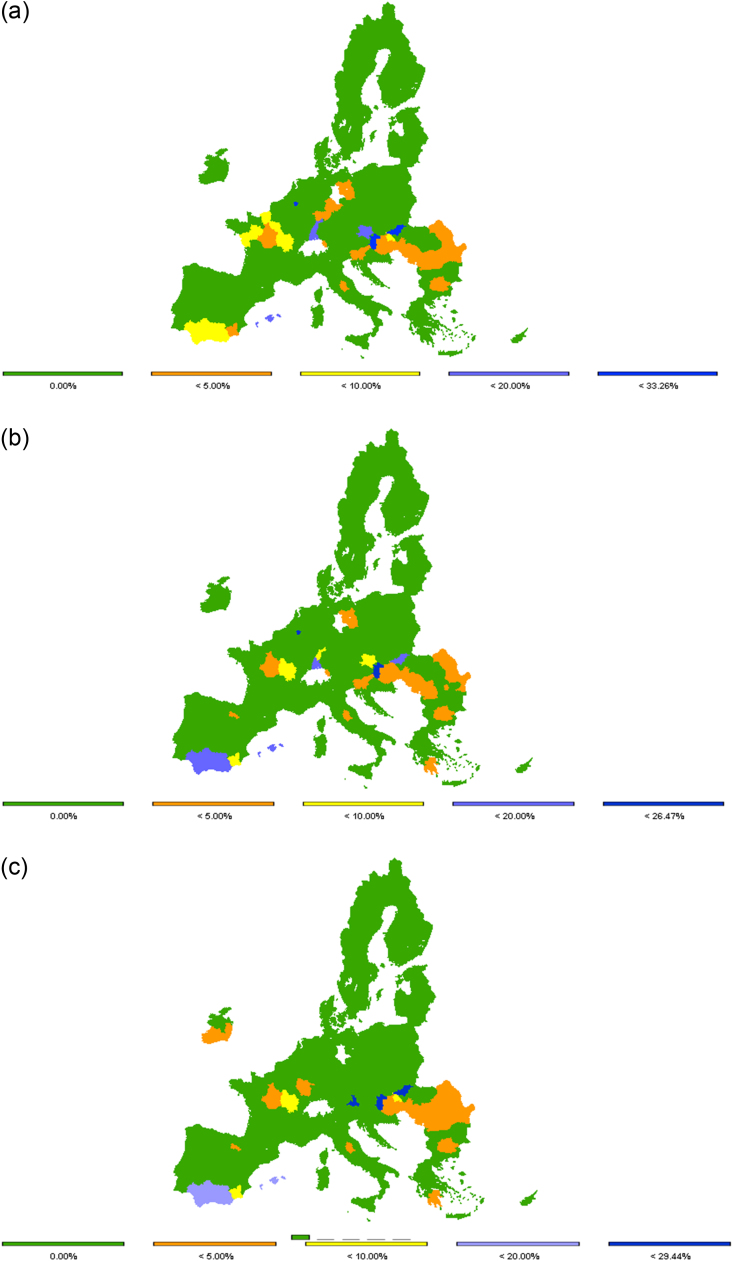
Actual reduction (relative changes to respective baseline) in water stress at NUTS 2 level in EU-27 in 2030 due to water reuse in REUSE (3a), WAVA REUSE (3b) and RAIN REUSE scenario (3c). Source: Own elaboration.

However, the actual situation is far from the identified potential (Fig. 2, Fig. 3). In the scenario REUSE, on average in the EU in 2030 the water stress reduction is only 1% compared to the potential 14%.^4^ Very few regions (Hungary, Bulgaria, Romania and Slovenia) actually use treated water and reduce freshwater use up to the identified potential (column 3 in Appendix C). Nevertheless, the potential or availability of treated water in these regions is very small compared to the total water resources (column 2 in Appendix C). On the other hand, Austria displays a high actual reduction in water stress mainly driven by area changes in sugar beet production. Due to higher average irrigated yields compared to rain-fed yields, there is a switch from a rain-fed to an irrigated area. With climate change, the water stress potential is declining because of projected increasing annual water availability and precipitation in central and northern European countries (Bisselink et al., 2018). Spain should not be neglected despite the identified low potential to reduce water stress. It can be noticed from the table in column 1 of Appendix B that Spain uses the largest quantities of treated water driven by an increase in olives for oil, which is around ¾ of the total treated water use in Europe, and it is doubling given the projected decline in water availability and precipitation due to climate change.

From Fig. 3a–c it is visible that, at NUTS 2 level, this is mainly occurring in the regions of Andalusia and Murcia, where there is a greater reduction in water stress compared to the national average in Fig. 2. The same can be noticed for some regions in France and Germany. However, in most of the Mediterranean regions, with a few exceptions in Spain and Italy, the actual water stress reduction is still marginal. Nevertheless, these regions rely considerably on irrigation and have great potential to reduce the water stress from agricultural production by using treated water. As long as there is no other incentive, such as a reduction in freshwater availability or changes in effective rainfall, farms rarely use treated water as an alternative supply. But this is again limited to some regions in the southern part of Europe where climate change is expected to have negative effects on water availability and precipitation. The actual water stress reduction in central and northern Europe is declining with climate change (Fig. 3b and c, compared to 3a) due to the positive effect on water resources, Still rendering the actual water stress reduction far from the identified potential.

### 4.2 Effect on agriculture

As expected, with more available water, farmers are switching from rain-fed to irrigated crop activities, leading to an increase in production. Columns 4 and 5 in Table 2 display the effect at EU level under the REUSE scenario. It may be noticed that, despite the switch in crop mix, overall production increases only marginally resulting in a small decline in producer prices for the most common produced crops. The crop-mix change is the result of a small decline in soft wheat and grain maize production (rain-fed crop variant) and an increase in durum wheat, sugar beet and olives for oil production (irrigated crop variant). Due to the high shares of rain-fed areas, the decline in cereals is greater than the increase in irrigated area. In addition, economic returns to these crops are relatively low therefore cannot afford to pay the price of treated water. On the contrary, irrigated production from rapeseed, sugar beet and olives for oil is increasing since they are less water intensive or value added crops. Nevertheless, due to the increase in irrigated agricultural production there will be an increase in production at aggregate level due to higher yields pushing down the prices. However, as a result of irrigated production, there is a noticeable increase in water use/costs which, together with negative producer prices, offset the higher production from the irrigated crop variant, resulting in insignificant income changes. The reduction in the relative profitability of irrigated crops compared to rain-fed crops also explains why there is a small production increase. As previously argued, despite the large available water quantities, production effect is limited due to the input cost for water. The positive production changes as well as income are mainly driven by the higher average yields from irrigated crop variants.

**Table 2:** Effects on crop-specific irrigated/rain-fed areas, yields, water use, prices and income in EU in 2030 under REUSE scenarios (relative % changes to respective baseline).

	Production (1 000 t)			Yield (kg/ha)			Water use (€/ha)	Producer price (€/t)	Income (€/ha)
	Aggr.	Rain-fed crop variant	Irrigated crop variant	Aggr.	Rain-fed crop variant	Irrigated crop variant			
Soft wheat	− 0.01	− 0.08	1.61	0.02	0.02	− 0.13	2.82	− 0.02	− 0.04
Durum wheat	0.41	− 0.90	12.22	0.47	− 0.04	2.15	16.15	− 0.06	0.35
Barley	0.01	− 0.12	2.54	0.05	0.06	− 0.38	3.69	− 0.02	0.00
Grain maize	− 0.01	− 0.21	0.48	0.06	− 0.03	0.07	0.35	− 0.01	0.03
Paddy rice	0.02	–	0.02	0.00	–	0.00	0.03	− 0.01	0.02
Rape seed	0.00	− 0.05	1.90	0.02	0.00	0.19	2.25	− 0.01	0.01
Sunflower	0.00	− 0.13	1.20	0.05	− 0.01	0.11	1.10	− 0.02	0.00
Soya	0.07	− 0.22	1.65	0.12	0.02	0.11	2.57	− 0.03	0.08
Sugar beet	0.11	− 0.42	4.02	0.21	0.09	0.09	4.40	− 0.06	0.44
Potatoes	0.02	− 0.09	0.22	0.05	0.01	0.01	0.30	− 0.05	− 0.06
Tomatoes	0.00	− 0.01	0.01	0.00	0.00	− 0.01	0.01	0.00	0.00
Other veget.	0.01	− 0.05	0.09	0.02	− 0.01	0.02	0.04	− 0.04	− 0.04
Apples	0.01	− 0.01	0.04	0.01	0.01	− 0.02	0.07	− 0.01	0.00
Other fruits	0.02	− 0.02	0.06	0.02	0.01	− 0.02	0.10	− 0.02	0.01
Citrus fruits	0.05	− 0.09	0.15	0.04	0.06	− 0.01	0.17	− 0.01	0.04
Table grapes	0.00	0.00	0.00	0.00	0.00	0.00	0.00	0.00	0.00
Olives for oil	0.47	− 0.50	1.57	0.49	− 0.13	0.41	1.20	− 0.09	0.45
Table olives	0.03	− 0.04	0.12	0.04	− 0.01	0.05	0.08	− 0.04	− 0.03

Source: Own elaboration.

Table 3, Table 4 show that the results are similar under WAVA REUSE and RAIN REUSE scenarios to the REUSE scenarios. The magnitude of the impact on production and water use differs and is slightly higher given the decline in freshwater availability for irrigation or decline in effective rainfall due to climate change. As previously noted, treated water becomes a slightly more important alternative source of irrigation for some regions (Fig. 3b and c) when there is an induced change in freshwater availability (water scarcity) or rainfall due to climate change. The increase in irrigated crop supply, the income and thus production effect are in general limited again to similar regions as well as crops as before, due to high water costs. It seems that demand elasticities in the model lead to a failure in the translation of higher crop production (water) costs into higher producer prices to stimulate production.

**Table 3:** Effects on crop-specific irrigated/rain-fed areas, yields, water use, prices and income in EU in 2030 under WAVA REUSE scenarios (relative changes to respective baseline).

	Production (1 000 t)			Yield (kg/ha)			Water use (€/ha)	Producer price (€/t)	Income (€/ha)
	Aggr.	Rain-fed crop variant	Irrigated crop variant	Aggr.	Rain-fed crop variant	Irrigated crop variant			
Soft wheat	− 0.01	− 0.10	2.15	0.03	0.04	− 0.57	5.61	− 0.03	− 0.06
Durum wheat	0.82	− 1.58	24.67	0.93	0.02	3.66	39.01	− 0.12	0.68
Barley	0.02	− 0.11	2.48	0.06	0.09	− 0.78	4.53	− 0.02	− 0.02
Grain maize	− 0.01	− 0.17	0.37	0.05	− 0.02	0.03	0.33	− 0.01	0.00
Paddy rice	0.05	–	0.04	0.01	–	0.01	0.04	− 0.01	0.05
Rape seed	0.01	− 0.04	1.59	0.02	0.00	0.16	1.94	− 0.01	0.00
Sunflower	0.03	− 0.16	1.80	0.10	0.00	0.23	1.82	− 0.04	0.01
Soya	0.07	− 0.27	1.79	0.14	0.03	0.07	2.67	− 0.03	0.08
Sugar beet	0.22	− 0.34	4.31	0.33	0.21	0.07	6.24	− 0.05	0.78
Potatoes	0.02	− 0.04	0.13	0.04	0.03	0.00	0.33	− 0.04	− 0.03
Tomatoes	0.00	0.00	0.01	0.00	0.00	− 0.01	0.01	0.00	0.00
Other veget.	0.01	− 0.03	0.06	0.01	0.00	0.01	0.03	− 0.02	− 0.02
Apples	0.01	− 0.01	0.06	0.02	0.01	− 0.02	0.10	− 0.01	0.01
Other fruits	0.04	− 0.03	0.11	0.04	0.01	0.00	0.15	0.00	0.01
Citrus fruits	0.10	− 0.19	0.33	0.09	0.10	0.01	0.34	− 0.03	0.10
Table grapes	0.00	0.00	0.00	0.00	0.00	0.00	0.01	− 0.03	0.00
Olives for oil	1.14	− 1.18	3.86	1.17	− 0.28	0.97	3.02	− 0.20	1.10
Table olives	0.07	− 0.10	0.28	0.09	− 0.03	0.11	0.20	− 0.09	− 0.04

Source: Own elaboration.

**Table 4:** Effects on crop-specific irrigated/rain-fed areas, yields, water use, prices and income in EU in 2030 under RAIN REUSE scenarios (relative changes to respective baseline).

	Production (1 000 t)			Yield (kg/ha)			Water use (€/ha)	Producer price (€/t)	Income (€/ha)
	Aggr.	Rain-fed crop variant	Irrigated crop variant	Aggr.	Rain-fed crop variant	Irrigated crop variant			
Soft wheat	− 0.01	− 0.11	2.49	0.03	0.05	− 0.58	6.19	− 0.03	− 0.07
Durum wheat	0.89	− 1.60	28.16	1.02	0.05	5.05	40.62	− 0.13	0.74
Barley	0.02	− 0.10	2.33	0.05	0.09	− 0.80	4.25	− 0.03	− 0.01
Grain maize	0.00	− 0.19	0.45	0.06	− 0.01	0.04	0.38	− 0.02	0.02
Paddy rice	0.05	–	0.04	0.01	–	0.01	0.04	− 0.01	0.05
Rape seed	0.01	− 0.03	1.57	0.01	0.00	0.19	1.89	− 0.01	0.00
Sunflower	0.03	− 0.17	1.89	0.10	0.00	0.25	1.88	− 0.05	0.01
Soya	0.06	− 0.21	1.63	0.11	0.02	0.05	2.63	− 0.03	0.09
Sugar beet	0.24	− 0.23	3.72	0.33	0.23	0.18	5.75	− 0.05	0.80
Potatoes	0.02	− 0.03	0.17	0.03	0.02	0.01	0.33	− 0.03	0.01
Tomatoes	0.00	0.00	0.01	0.00	0.00	− 0.01	0.01	0.00	0.00
Other veget.	0.00	− 0.01	0.02	0.00	0.00	0.00	0.03	− 0.01	− 0.01
Apples	0.02	− 0.02	0.07	0.02	0.02	− 0.02	0.13	− 0.01	0.01
Other fruits	0.05	− 0.03	0.14	0.06	0.02	0.01	0.19	0.00	0.02
Citrus fruits	0.12	− 0.21	0.36	0.11	0.12	0.00	0.38	− 0.03	0.11
Table grapes	0.00	0.00	0.00	0.00	0.00	0.00	0.01	− 0.04	0.00
Olives for oil	1.24	− 1.25	3.76	1.27	− 0.29	0.95	2.86	− 0.21	1.20
Table olives	0.08	− 0.10	0.28	0.09	− 0.02	0.11	0.19	− 0.10	− 0.07

Source: Own elaboration.

**Fig. 4:**
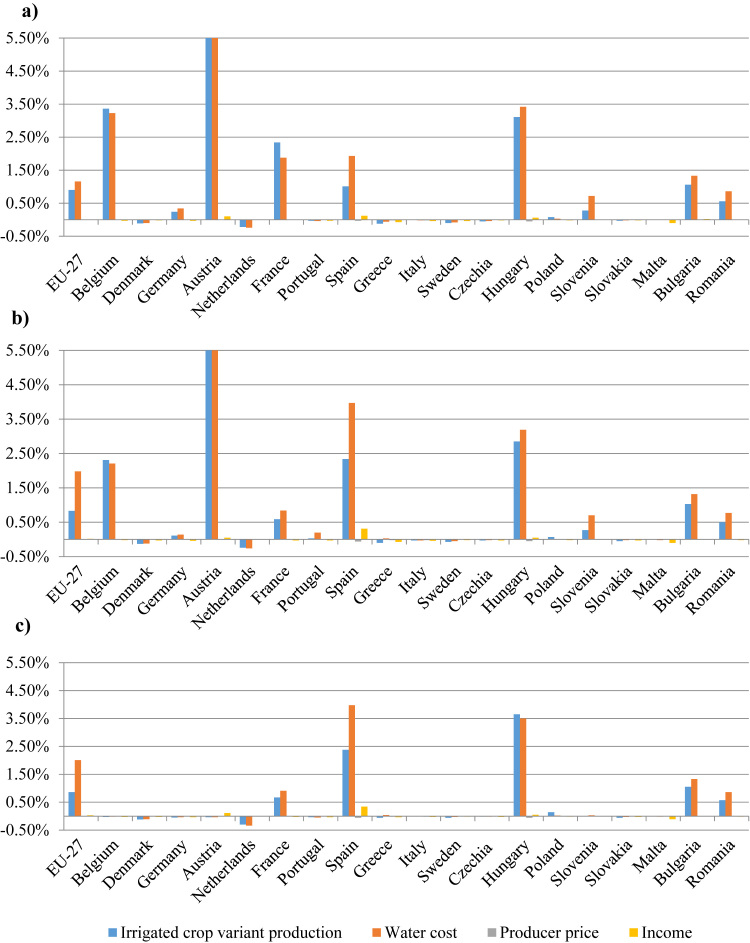
Effect of water reuse on irrigated crop production, water cost, price and income in 2030 under the REUSE (4a), WAVA REUSE (4b) and RAIN RESUE (4c) scenarios (relative changes to respective baseline) Note: Appendix D provides the actual numbers for producer prices and income. Source: Own elaboration.

Looking into country-level data, some nuance can be introduced to the statement that costs are an impediment to address water scarcity with water reuse as an alternative source (Fig. 4). When the impact of climate change leads to a reduction in freshwater availability, treated water does become a mitigation option, but only limited to Spain (Andalusia and Murcia regions more specifically). Recall that for the northern and central European countries the projected climate change effects on water availability and precipitation were beneficial. As a result, countries such as Belgium, Germany and France use less treated water for irrigation (Fig. 4b) or do not even irrigate at all, like in the example of Belgium and Austria in the RAIN REUSE scenario (Fig. 4c). In the case of Spain, the increase in irrigated area/supply is mainly due to the use of this alternative water source for growing olives for oil. However, the increase in associated water costs is even higher. Thus, irrespective of the small increase in production compared to the baseline, the increase in water cost combined with the decline in producer prices due to higher supply results in very small income changes. These results display an important message, namely that under a less available water for irrigation or rain scenario, treated water is an alternative supply option which, without impacting farmers’ income, can reduce the water stress from agricultural production. However, the potential of the treated water is not fully exploited if the costs of treated water are lower those farmers are paying for freshwater or if farmers obtain financial support in the form of subsidies to cover the treatment, transport, storage and investment costs.

## 5 Discussion

In this paper using agro-economic modelling, we are able to assess the potential of treated water to reduce freshwater abstraction and thus water stress and address water scarcity induced by climate change. Using the CAPRI model and data on treated water quantities at NUTS 2 level, we showed that treated water is a possible alternative supply source to address water shortages with very negligible effects on farmers’ income and food production in the EU. Food production will change marginally and be slightly more dependent on irrigated agriculture. Our findings that treated water can contribute to water stress reduction in Spain, Germany and France corroborate with those from Lazarova et al. (2001), Barbagallo et al. (2012), Pedrero et al. (2010), but not with Vergine et al. (2017) in the case of Italy. In addition, the estimated potential is also in line with that identified by Sanz and Gawlik (2014), with few exceptions such as Belgium, the Netherlands and Italy. Pistocchi et al. (2017) calculated an average reduction of around 11% per year in the EU.

However, the actual water reuse and water stress reduction is far from the identified potential (14% of total available water) and is only limited (1% of total available water or 7% of total available treated water) to several regions across the EU (Spain, Austria, France, Germany). This is in line with previous studies (European Commission, 2018a), where it is identified that the current uptake of treated water (1.7 billion m^3^) falls far below its full potential (6.6 billion m^3^) without any EU legal framework imposed.

In our study, the high price (€0.5/m^3^) equal to the total treatment cost (full cost recovery) is the reason for such a low implementation.^5^ Climate change effects on water availability and precipitation even failed to induce higher use across the EU (see Fig. 2 and Appendix C). Under these scenarios, the reduction in the pressure on the water resources increases marginally compared to the REUSE alternative scenario and is still far from the identified potential. The inclusion of relevant externalities may have a strong impact on the economic feasibility of using treated water (Garcia and Pargament, 2015). Thus, unless farmers consider the environmental benefits of treated water in their decision-making, they will always find surface water more profitable than treated water (Alcon et al., 2013). To test the potential impact of water costs being borne by other agents, we conduct a sensitivity analysis considering different cost recovery scenarios (50%, 20% and 15% of the access cost). However, we obtained similar results with a reduction in water stress of around 1% when treated water was priced at €0.5/m^3^. This is not surprising given that farmers in the EU pay a small share of the total irrigation cost, with average prices sometimes well below the treatment cost, ranging between €0.005/m^3^ in Germany to €0.12/m^3^ in Italy (Berbel et al., 2019). The main reason that could explain why our findings for Italy differ from those of Vergine et al. (2017). Spanish farmers pay more (€0.12/m^3^ and €0.24/m^3^ on average over the last 10 years) and are willing to pay more than double to ensure water supply (Alcon et al., 2014). This is in line with our findings where we noticed the highest amount of treated water use given the cost of €0.5/m^3.^

It should be noted that the simulation results are subject to some limitations and qualifications. As previously pointed out, in our modelling exercise we are not able to separate treated water from freshwater in the CAPRI model. However, even if we were able to model properly, as Alcon et al. (2013) argue, farmers will always give priority to freshwater or groundwater without considering any non-market benefits in their decision-making. This is not the case in CAPRI. In addition, deficit irrigation is not explicitly modelled. However, even if considered as an adaptive strategy in dry regions, our results will not change much; this is mainly because, firstly, CAPRI does not consider crop growth stages and, secondly, the water saving from deficit irrigation will actually mean less freshwater or treated irrigated water requirements. Hence, the consumed treated water quantities may be considered as the maximum quantities that can be consumed. Furthermore, the CAPRI water database, being from 2006, should not be seen as an impediment. As pointed earlier, the main driver for the simulation results is the high water price imposed in both the baseline and the alternative scenarios. Updating it to more recent data from LISFLOOD is desirable but this will not make any difference in the current exercise given the inability to separate treated water, the imposed high water price or not capturing non-market benefits during the optimisation. Last, our modelling approach considers water availability at NUTS2 level. This approach fails to fully capture the fact that water uses, costs and availability, as water networks are much more local. However, the CAPRI model is designed to represent whole regions instead single farms or fields. Hence, there is a distribution of cost functions in the regions that are embedded in a single function and a regional water price of €0.5/m^3^ should be interpreted as an average price despite the costs are not uniform in reality. Therefore, even though an increase in irrigated area of 1 ha at an average price of €0.5/m^3^ looks unrealistic, the predicted area also cover the part of the distribution where farmers irrigate at much lower costs. The assumptions on the Positive Mathematical Programming (PMP) terms of the quadratic cost function to allow smooth substitution between rain-fed and irrigated crop variants, also drives to some extent the model response. Meaning, it implies that when simulating high water prices, the model may show small irrigated areas in regions where irrigation is not expected at all. Therefore, our results may partly overestimate the potential, in spite of the small, use of reused water even when the cost estimates from Pistocchi et al. (2017) take into account the deployed of the water from the waste treatment plant to the field, including all costs of infrastructure, treatment and pumping.

## 6 Conclusion

In this paper we use an agro-economic model to see the uptake of the theoretical availability of treated water for irrigation. Our results show that considering the cost associated with making available treated water to farmers the use of this water source is limited. Despite the caveats discussed above, as well as the non-conventional way of calculating the water stress, we believe that our results are realistic in this first attempt to model treated water in an agricultural economic model. The results help policy makers and stakeholders to obtain a better understanding of the role water reuse may have as a future adaptation measure for water scarcity and climate change. Together with the recently adopted regulation on minimum quality requirements and the risk management of using treated water, feasible policy measures can be proposed that can complement the environmental objectives of the CAP. Nevertheless, in order to promote the sustainable management of water resources and the full potential of treated water is to be exploited, financial support is required in the form of subsidies to cover the treated water price that reflects the treatment, transport, storage and investment cost. According to the European Council (2020b, p. 7) ʻfinancial incentives for practicing water reuse in agriculture have been identified as being among the reasons for the low uptake of water reuse in the Unionʼ. However, the one-size-fits-all approach via a flat rate water price is not desirable. From our results it becomes apparent that a flat rate of €0.5/m^3^ can only lead to a full absorption of the supplied treated water resource by farmers in a limited number of member states. Thus, a preferable option for agricultural irrigation would be a ʻfit-for-purposeʼ approach, i.e. an approach that will maximise the potential of reused water to address water scarcity, and able to provide a higher volume of treated wastewater at lower cost. The impact assessment analysis by the European Commission (2018b) also concluded that this is a preferred option.

This ʻfit-for-purposeʼ approach is very relevant in the long term. Despite the positive climate change effects on water resources in central and northern Europe, drought events are present and may become more persistent in time, thus water reuse will become more important. However, when these become recurrent and affect the availability of freshwater stored in reservoirs, the farming sector will possibly be even more dependent on alternative water resources.

Our results also inform and support the need for consistency between the two existing water policies: the Water Framework Directive and the Urban Waste Water Treatment Directive. These policies identify and encourage the reuse of treated water, but specific conditions are not present (European Commission, 2018a). Ensuring coherent conditions within the existing EU legislative frameworks on water will ensure the applicable levels of environmental protection and food security are maintained. However, these conditions should also be in line with the ʻfit-for-purposeʼ approach. However, given the transboundary issue of the river basin aspect in the WFD, proposing different rates in different MS should also be carefully defined and chosen.

Finally, the results may also be relevant and linked in the future to other technological alternatives such as a ʻseawater greenhouseʼ based on thermally evaporating seawater or water from desalination based on photovoltaic energy and reverse osmosis. Separating freshwater and alternative irrigation water sources to see how the alternatives displace (or do not displace) the current water available for irrigation or allow the expansion of irrigation to previously rain-fed areas is something that we would like work on and investigate in the future.

## Funding

This research did not receive any specific grant from funding agencies in the public, commercial or not-for-profit sectors.

## Disclaimer

The views expressed are purely those of the authors and may not in any circumstances be regarded as stating an official position of the European Commission.

## Declaration of Competing Interest

The authors declare that they have no known competing financial interests or personal relationships that could have appeared to influence the work reported in this paper.

## Figures and Tables

**Table:** 

CAPRI crop activities	CROPWAT crop activities	WOFOST crop activities
Soft wheat	Soft wheat	Soft wheat
Durum wheat	Soft wheat	Soft wheat
Rye	Soft wheat	Rye
Barley	Soft wheat	Barley
Oats	Soft wheat	Soft wheat
Maize	Maize	Maize
Other cereals	Soft wheat	Rye
Rapeseed	Sunflower	Rapeseed
Sunflower	Sunflower	Sunflower
Soya	Sunflower	Sunflower
Fodder maize	Maize	Maize
Fodder root crops	Maize	Maize
Other fodder crops	Maize	Maize
Extensive grass production	Maize	Maize
Intensive grass production	Maize	Maize
Paddy rice	Paddy rice	Paddy rice
Olives for oil	Olives for oil	Soft wheat
Pulses	Maize	Pulses
Potatoes	Potatoes	Potatoes
Sugar beat	Sugar beat	Sugar beat
Tobacco	Sugar beat	Sugar beat
Tomatoes	Tomatoes	Potatoes
Other vegetables	Tomatoes	Potatoes
Apples	Apples	Potatoes
Other fruits	Apples	Potatoes
Citrus fruits	Citrus fruits	Potatoes
Table grapes	Table grapes	Soft wheat
Table olives	Olives for oil	Soft wheat
Wine production	Wine production	Soft wheat

**Table:** 

Region	Available treated water at 0.5 €/m^3^ (1000 m^3^)	Proportion relative to total available irrigated water in BASE and RAIN scenario^a^ (%)	Proportion relative to total available irrigated water in WAVA scenario (%)
EU-27	6,592,143	13.5	13.6
Belgium	4812	34.3	31.4
Denmark	49,628	10.8	10.2
Germany	110,002	16.8	14.9
Greece	262,662	4.3	4.4
Spain	2,054,378	10.1	10.5
France	585,456	12.2	11.5
Italy	2,968,893	32.5	32.1
Netherlands	59,468	24.6	23.1
Austria	16,311	17.8	15.8
Portugal	419,671	9.4	10.2
Sweden	4211	3.3	3.0
Czechia	985	1.5	1.3
Hungary	14,493	3.2	3.0
Poland	3643	2.8	2.2
Slovenia	11	0.7	0.7
Slovakia	23,264	10.1	9.0
Malta	2105	7.4	7.3
Bulgaria	5082	1.2	1.2
Romania	7069	0.9	1

a Note that the water availability in RAIN scenario is the same as in the BASE. Recall that in RAIN scenario only the effective rainfall is affected in simulation year but water availability is not affected.

**Table:** 

	REUSE scenario			WAVA REUSE scenario			RAIN REUSE scenario		
Region	Actual use of treated water (1000 m^3^)	Proportion relative to total available irrigated water in BASE scenario (%)	Proportion relative to total available treated water (%)	Actual use of treated water (1000 m^3^)	Proportion relative to total available irrigated water in WAVA scenario (%)	Proportion relative to total available treated water (%)	Actual use of treated water (1000 m^3^)	Proportion relative to total available irrigated water in RAIN scenario (%)	Proportion relative to total available treated water (%)
EU-27	433,523	0.78	6.58	747,206	1.36	11.33	764,671	1.38	11.60
Belgium	417	2.21	8.66	295	1.46	6.13	–	–	–
Denmark	–	–	–	–	–	–	–	–	–
Germany	2209	0.29	2.01	945	0.11	0.86	–	–	–
Greece	–	–	–	1198	0.02	0.46	1644	0.03	0.63
Spain	341,350	1.53	16.62	681,356	3.14	33.17	714,378	3.20	34.77
France	57,883	1.07	9.89	26,978	0.48	4.61	26,684	0.50	4.56
Italy	–	–	–	–	–	–	–	–	–
Netherlands	–	–	–	–	–	–	–	–	–
Austria	12,254	11.33	75.12	6824	5.70	41.84	–	–	–
Portugal	–	–	–	7112	0.16	1.69	–	–	–
Sweden	–	–	–	–	–	–	–	–	–
Czechia	–	–	–	–	–	–	–	–	–
Hungary	13,368	2.89	92.23	13,385	2.66	92.36	13,385	2.90	92.36
Poland	34	0.03	0.94	5	0.00	0.14	14	0.01	0.39
Slovenia	11	0.72	97.55	11	0.69	100	1	0.03	4.56
Slovakia	–	–	–	–	–	–	–	–	–
Malta	–	–	–	–	–	–	–	–	–
Bulgaria	5010	1.21	98.59	5002	1.19	98.44	5005	1.20	98.49
Romania	7069	0.84	100.00	6871	0.76	100.00	7069	0.84	100

**Table:** 

	REUSE scenario		WAVA REUSE scenario		RAIN REUSE scenario	
Region	Prices (%)	Income (%)	Prices (%)	Income (%)	Prices (%)	Income (%)
EU-27	-0.01	0.01	-0.01	0.02	-0.01	0.03
Belgium	-0.01	-0.03	-0.01	-0.02	-0.01	-0.03
Denmark	-0.01	-0.02	-0.01	-0.03	-0.01	-0.03
Germany	-0.01	-0.03	-0.01	-0.04	-0.01	-0.04
Greece	-0.01	0.10	-0.02	-0.07	-0.02	-0.04
Spain	0.00	-0.01	-0.06	0.31	-0.06	0.34
France	-0.01	0.00	-0.01	-0.03	-0.01	-0.03
Italy	-0.01	-0.03	-0.02	-0.04	-0.02	-0.03
Netherlands	-0.03	0.12	0.00	-0.01	0.00	-0.01
Austria	-0.02	-0.07	-0.01	0.05	-0.01	0.11
Portugal	-0.02	-0.04	-0.01	-0.03	-0.01	-0.04
Sweden	-0.01	-0.04	-0.01	-0.02	-0.01	-0.02
Czechia	0.00	-0.02	-0.01	-0.03	-0.01	-0.03
Hungary	-0.05	0.06	-0.05	0.05	-0.06	0.05
Poland	0.00	-0.02	0.00	-0.02	0.00	-0.02
Slovenia	-0.01	-0.01	-0.01	-0.01	-0.01	-0.01
Slovakia	-0.01	-0.02	-0.01	-0.03	-0.01	-0.03
Malta	-0.01	-0.10	-0.01	-0.10	-0.01	-0.11
Bulgaria	0.00	0.02	-0.01	0.00	-0.01	0.00
Romania	-0.01	-0.01	-0.01	-0.02	-0.01	-0.02

## Footnotes

1 www.fao.org/land-water/databases-and-software/cropwat/en/.

2 www.wur.nl/en/Research-Results/Research-Institutes/Environmental-Research/Facilities-Products/Software-and-models/WOFOST.htm.

3 Potentially irrigated area compared to actual is the maximum area which could be irrigated using the equipment and the available water quantity. The total irrigable area may differ from the actual since the equipment may be mobile and therefore utilisable on several fields depending on the available water quantities.

4 Appendices B and C provide the actual numbers for both potential and actual water stress.

5 While it could be argued that following the polluter pays principle, treatment costs could be covered by the polluter (industrial or residential users) the lion’s share (42 euro cents) of the total cost comes from making water accessible from the waste treatment plant to the field.
